# Clustering-Triggered Emission of Carboxymethylated Nanocellulose

**DOI:** 10.3389/fchem.2019.00447

**Published:** 2019-06-20

**Authors:** Meng Li, Xiaoning Li, Xuefei An, Zhijun Chen, Huining Xiao

**Affiliations:** ^1^Hebei Key Lab of Power Plant Flue Gas Multi-Pollutants Control, Department of Environmental Science and Engineering, North China Electric Power University, Baoding, China; ^2^MOE Key Laboratory of Resources and Environmental Systems Optimization, Ministry of Education, Beijing, China; ^3^Key Laboratory of Bio-based Material Science and Technology of Ministry of Education, Northeast Forestry University, Harbin, China; ^4^Department of Chemical Engineering, University of New Brunswick, Fredericton, NB, Canada

**Keywords:** carboxymethylated nanocellulose, clustering-triggered emission, carboxyl groups, space conjugation, nanomaterials

## Abstract

Non-conjugated polymers with luminescence emission property have recently drawn great attention due to their promising applications in different areas. Most traditional organic synthetic non-conjugated polymers required complicated synthesis. Herein, we report a non-conjugated biomass material, carboxymethylated nanocellulose (C-CNC), which is found to be practically non-luminescent in dilute solutions, while being highly emissive when aggregated as nanosuspensions. We propose that the luminescence of C-CNC originates from the through-space conjugation of oxygen atoms and carboxyl groups of C-CNC. Thus, a clearer mechanism of clusteroluminescence was provided with the subsequent experiments. The effects of concentration of C-CNC, solvent, temperature and pH have also been investigated. In addition, ethylenediamine (EDA) has been employed to “lock” C-CNC material via the bonding of amide groups with carboxylic groups. As prepared C-CNC/EDA confirmed that the clusteroluminescence was attributed to the amide moieties and through-space conjugation between oxygen and carbonyl moieties. Density functional theory (DFT) calculations have also been employed to confirm the luminescence mechanism. It is believed that such clustering-triggered emission mechanism is instructive for further development of unconventional luminogens.

## Introduction

Over several decades, fluorescent organic molecules and nanomaterials have attracted much attention and interest due to their special photophysical properties and wide applications in areas such as bioimaging, organic light-emitting diodes (OLEDs), light-emitting electrochemical cells (LECs), and phototherapy reagents, etc (Schwartz et al., 2007; Qin et al., 2012; Wang et al., 2012; Cheng et al., 2015; Li et al., 2018c). Initially, fluorescent organic molecules are widely employed in inorganic ion detection, drug analysis, etc (Bian et al., 2014; Li et al., 2018b). However, most of the reported probe molecules have suffered from the aggregation-caused quenching (ACQ) effect and faced the problem of poor solubility, biocompatibility, and solid-state luminescence, which has greatly limited their potential applications (O'Brien et al., 2010; Wu and Butt, 2016; Huang et al., 2019). Until 2001, Tang and co-workers raised the concept of aggregation-induced emission (AIE), and reported AIE luminogens (Luo et al., 2001). Compared to ACQ molecules, AIE luminogens could turn from non-luminescent or weak fluorophors into strong emitters when they are aggregated into nanoparticles in poor solvents or fabricated into thin films in solid state (Xing et al., 2007; Yamaguchi et al., 2017). Therefore, many AIE luminogens have been synthesized to understand the luminous mechanism due to their unique luminescence (Chen et al., 2013; Qiu et al., 2018). Most AIE luminogens are generally constructed by aromatic groups and conjugated subunits, which function as chromophore centers (Yuan et al., 2014). However, achieving organic fluorescent nanomaterials with aggregation-induced emission is not an easy task as lots of aggregation-induced emission luminogens required complicated synthesis or toxic starting materials.

Recently, some non-conventional luminogens such as non-conjugated polymers, which are free of aromatic building blocks, have been reported due to their unique clustering-triggered emission (CTE) (Yuan and Zhang, 2017; Chen et al., 2018a). Compared to the conventional AIE luminogens, the CTE luminogens are generally more hydrophilic, easy to prepare, and biocompatible, making them ideal for biological and medical applications (Qin et al., 2017; Dou et al., 2018). Typically, these polymers have electron-rich heteroatoms, such as nitrogen (N), oxygen (O), sulfur (S), phosphorus (P) (Zhao et al., 2015; Zhou et al., 2016; Shang et al., 2017). Therefore, the luminescence mechanism may be attributed to clustering of these electronics-rich atoms and subsequent electron cloud overlaps to form extend conjugation to rationalize the unique AIE properties (Shen et al., 2012; Zhu et al., 2015). Moreover, it is found that some natural products also have visible light in concentrated and solid state conditions, such as dendrimers, starch, amino acids and sodium alginate, etc (Wang and Imae, 2004; Ye et al., 2017; Dou et al., 2018; He et al., 2018). Such CTE materials have huge potential in sensing and cell imaging technologies, which could contribute to the development of nano-luminescence materials (Jin et al., 2016; Ma et al., 2016; Li et al., 2017).

Most clustering-triggered emission (CTE) luminogens have been fabricated by precipitation of precursor organic molecules and polymers (Mohamed et al., 2015; Li et al., 2017; Lu et al., 2017). However, few works have explored the luminescence of natural products with low cost and good biocompatibility (Zhao et al., 2016). In this work, we studied the luminescence behavior of carboxymethylated nanocellulose (C-CNC) with rich carboxyl groups (Dong and Roman, 2007; Yang et al., 2016). The as-prepared C-CNC showed excitation/polarity-dependent fluorescence emission, indicating their potential in differentiating solvents with different polarities. Additionally, the effects of concentration of C-CNC, solvent, temperature, pH and amination with EDA have been investigated. Density functional theory (DFT) calculations have also been employed to understand the clusteroluminescent emission that could be attributed to through-space conjugation between oxygen atoms and carbonyl groups (Miao et al., 2016; Zhu et al., 2016). These results should be highly implicative for further understanding the emission of CTE luminogens.

## Experimental Section

### Chemicals and Instruments

The experimental reagents used in this research were isopropyl alcohol, methano, ethanol absolute, N,N-dimethylformamide, tetrahydrofuran, ethylenediamine, monochloroacetic acid, hydrochloric acid, degreased cotton, sodium chloride, sodium hydroxide, sodium carbonate, sodium perborate, and pH buffer. All these materials were AR grade and purchased from Tianjin Kermel Chemical Reagent. Ethanol absolute, N,N-dimethylformamide and tetrahydrofuran were analytical of chromatographic grade. The water used in all experiments was ultrapure water, produced using a Smart-RO ultrapure water system (Hitech Instruments Co., Ltd., Shanghai, China).

The instruments were used as follows: mass flow meter; F97Pro fluorescence spectrophotometer; DF-101S heat collection-constant temperature type magnetic stirrer, Mettler Toledo electronic balance; SHENGYANG Ultrasonic Cleaner, TH21 Heating thermostat.

### Characterization

The scanning electron microscopy (SEM) images on the C-CNC were observed with the magnification of 2500–100 000 times via the HitachiS-4800 SEM. After the samples were sonicated for 10–15 min and then dripped onto the conductive adhesive film, the acceleration voltage of this process was 0.5–30 kV. The crystal structure of the sample at an acceleration voltage of 200 kV was observed using a JEM-2100 transmission electron microscope (TEM).

The crystalline phase of the sample was characterized by X-ray diffraction (XRD, Bruker D8 advance) at a wide angle (10–90°) scan in steps of 0.01 degrees. And the functional group on the surface of C-CNC was measured by Fourier transform infrared spectrometer (FT/IR-200, JASCO, Japan), and for each sample, a scan of 50 mg of the sample in the range of 4,000–500 cm^−1^ was recorded with a wave number accuracy of 0.01 cm^−1^. The X-ray photoelectron spectrum (XPS) of the sample was measured on a MultiLab 2000 XPS (Thermo VG Scientific). ^1^H NMR spectra were recorded on an AVANCE III HD 500 MHz spectrometer (Bruker Corp., Karlsruhe, Germany) with D_2_O. Theoretical calculations were carried out using the B3LYP/6-31G(d) basis set and density functional theory (DFT). The fluorescence lifetimes of C-CNC were measured on a FLS980 spectrometer (Edinburgh Instruments, UK). The quantum yield (Φ) was determined by a FLS920 spectrometer with an excitation wavelength of 360 nm at 25°C (Edinburgh Instruments, UK). Dynamic light scattering (DLS) was measured on a ZEN3700 (Malvern, UK).

### The Preparation of C-CNC

C-CNC was synthesized according to the reported methods (Hebeish et al., 2010). Three gram finely degreased cotton was dispersed in 180 mL isopropanol and mechanically stirred at 25°C for 30 min. A calculated volume of 8 mL 50% NaOH (w/v) was added dropwise over about 10 min and the mixture was continuously stirred at 25°C for 60 min. Monochloroacetic acid (0.4 g) dissolved in 15 mL of isopropyl alcohol and then neutralized with the equivalent amount of Na_2_CO_3_. After the temperature rose to 60°C, the above mixture continued to stir for 60 min. At the end of this process, the mixture was filtered and suspended in 150 mL of 80% methanol and then neutralized with acetic acid. After the reaction, the sample was washed three times with methanol and dried to obtain C-CNC. Subsequently, C-CNC was purified by precipitation and freeze dried for further use. The carboxyl group content measured by conductivity was 0.6 mmol/g (Figure S1).

### The Preparation of C-CNC/EDA

C-CNC (100 mg) and EDA (4 μL) were dissolved in deionized water (50 mL) and mixed uniformly. Then the solution was transferred to a pressure bottle (100 mL) and heated at 100°C for 8 h in a vacuum oven. After the reaction, the reactors were cooled down to room temperature naturally. The prepared sample was directly measured using a fluorescence spectrophotometer.

## Results and Discussion

### Characterizations

**Figure 1:**
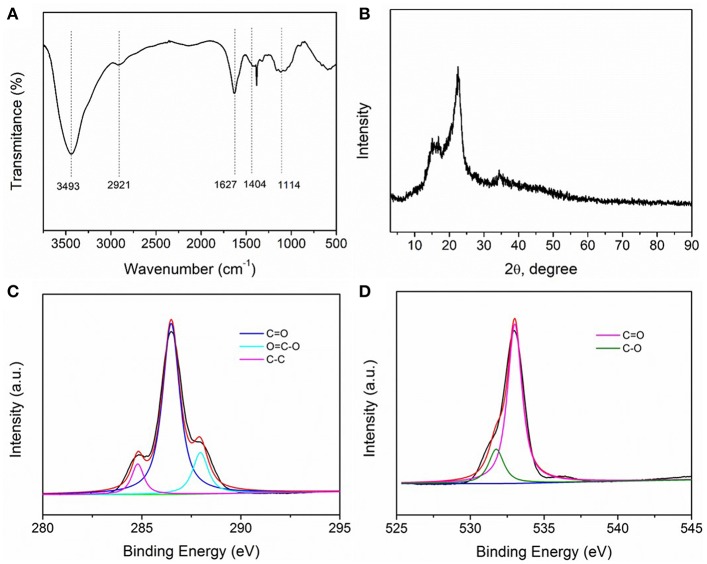
**(A)** FTIR spectra of C-CNC, **(B)** X-ray diffraction pattern of C-CNC, **(C)** C 1s XPS spectrum of C-CNC, **(D)** O 1s XPS spectrum of C-CNC.

Fourier Transform Infra-Red (FTIR) spectroscopy was initially employed to understand the structure of C-CNC (Figure 1A). A plurality of broad peaks in the 1,000–1,200 cm^−1^ region correspond to -C-O-C- stretching vibration. For the bending vibration mode of -CH_2_, the spectrum of C-CNC showed a peak at 2,921 cm^−1^. The broad peaks in the 3,200–3,600 cm^−1^ region correspond to -OH stretching vibrations. For C-CNC, the main spectra of the peaks at about 1,404 cm^−1^ corresponds to COO- symmetrical stretching vibrations and the peaks at about 1,627 cm^−1^ ascribed to the asymmetric stretching vibrations of COO-. A peak at 3,493 cm^−1^ appeared in the spectrum, ascribed to the -OH stretching vibrations for C-CNC.

As shown in Figure 1B, XRD characterization of the C-CNC was performed to investigate the phase structure of the sample. The diffraction peaks of C-CNC at 15.1°, 16.8°, 22.5°, and 34.3° corresponding to the (1-10), (100), (200), (004) crystallographic planes, which indicates the C-CNC belongs to the typical type of cellulose I (Li and Renneckar, 2011; Rafieian and Simonsen, 2014; Du et al., 2016). XPS measurement was examined to further study the chemical state changes of the C-CNC. It can be seen from Figure 1C that the high-resolution C 1s XPS spectra of the sample. For the C-CNC, the peak at 288.9 eV (C1) is typically ascribed to the sp^2^-bonded carbon (O-C = O). The peak at 287.8 eV (C2) is considered to C = O bond. And the peak at 284.6 eV (C3) could be ascribed to sp^2^ C-C bonds. These results confirm the existence of carboxyl groups in C-CNC. The XPS O 1s spectra can be seen from Figure 1D with peaks at 530.78 eV and 532.88 eV, corresponding to C-O and C = O bond in the carboxyl group of C-CNC (You et al., 2017). A representative SEM image of the C-CNC can be seen from Figure S2a, which shows a rugged surface (Querejeta-Fernandez et al., 2014). Figure S2b shows a typical transmission electron microscope (TEM) image of C-CNC. The C-CNCs in water show a substantially clear dispersion with a rod-like morphology (Habibi et al., 2010; Majoinen et al., 2014). The Raman spectrum of C-CNC presents that there is no visible raman peaks with large slits of C-CNC, which may be due to the poor fluorescent intensity of C-CNC solutions (Figure S3).

### Fluorescent Tests of C-CNC

As a starting point for our research, excitation-dependent fluorescence emission of C-CNC was observed (Figure S4). We could see that the bathochromic shift in fluorescence when the excitation wavelength was increased from 320 to 360 nm. Additionally, it found a slight difference at 320 nm, which might be attributed to the cluster size of C-CNC. Upon addition of organic solvent, C-CNC might form aggregates in different sizes. Aggregates in different size might have different through-space conjugated structures, which made these aggregates with different excitation and emission wavelengths. Additionally, these through-space conjugated structures had a high molecular energy gap and were mainly located in deep UV region, as they cannot form a big conjugated area. As a result, exposure of the sample to different wavelengths meant different energy-matched aggregates would be excited. However, the shape of fluorescence emission did not substantially change with excitation wavelength for normal molecularly dispersed organic chromophores. This result might be attributable to polydisperse through-space-conjugation units consisting of carbonyl moieties (Dou et al., 2018; Ma et al., 2018). Moreover, the 360 nm was selected as the excitation wavelength with its nice emission intensity in the subsequent experiments.

**Figure 2:**
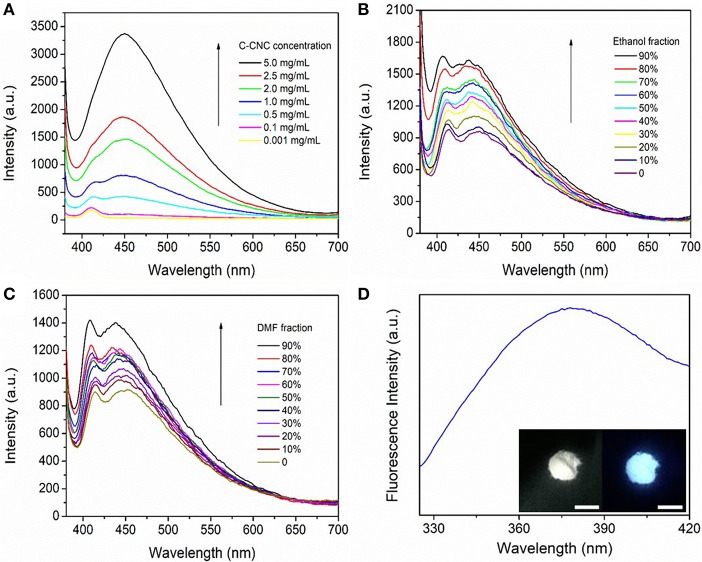
**(A)** Fluorescence emission of C-CNC at different concentrations in water (excitation wavelength = 360 nm), **(B)** Fluorescence emission of C-CNC (0.5 mg/mL) in mixtures of ethanol and water (excitation wavelength = 360 nm), **(C)** Fluorescence emission of C-CNC (0.5 mg/mL) in mixtures of DMF and water (excitation wavelength = 360 nm), **(D)** Fluorescence emission of C-CNC in the solid state (excitation wavelength = 300 nm). Inset: images of C-CNC powder in bright field and upon excitation at 365 nm. Scale bar = 1 cm.

To get insight into the clustering of C-CNC, the absorption spectra of different concentrations of C-CNC were examined (Qin et al., 2017; Chen et al., 2018b). At low concentrations, we could only see the single maximum absorption peak of C-CNC. With the concentration of C-CNC slowly increasing, a new red-shifted peak appears at 340 nm (Figure S5a) (Ma et al., 2018). When the C-CNC reached an optimum concentration of 0.5 mg/mL, the peak intensity increased significantly (Figure S5a). These results provide a likelihood for the presence of aggregation in concentrated solutions of C-CNC (Chen et al., 2018b). And the apparent peak of C-CNC around 340 nm was found under excitation (Figure S5b). As shown in Figure 2A, there was a similar trend between fluorescence and UV-vis spectra of C-CNC solutions. Enhancement of fluorescence was also observed when the concentration of C-CNC was increased, with higher concentrations triggering constant fluorescence enhancement. When the C-CNC concentration reached 5.0 mg/mL, the fluorescence was significantly enhanced, indicating a positive correlation and fluorescence emission of C-CNC (Figure S5c). It can be seen from Figure 2B that the fluorescence was very low when C-CNC was dissolved in good solvent water. However, the fluorescence intensity gradually increased, as the poor solvent ethanol was added to the C-CNC aqueous solution (Zhou et al., 2016; Qin et al., 2017; Yang et al., 2017). When the fraction of ethanol is 90%, the fluorescence of C-CNC is increased by 1.6 times, which provides conclusive evidence that C-CNC produces cluster-induced luminescence. Moreover, other organic solvents such as N,N-dimethylformamide (DMF) and tetrahydrofuran (THF) had also been employed to verify the clustering-triggered emission. The C-CNC showed fluorescence enhancement as the DMF and THF concentration increased, indicating an interaction between the fluorophore and the solvent (Figures 2C and S5d), namely, the polarity of the solvent affected the fluorescence emission (Zhao et al., 2015; Miao et al., 2016; Gu et al., 2018). C-CNC showed strong fluorescence emission in the solid state with a quantum yield of 7.8% (Figure 2D), meanwhile, the C-CNC aqueous solution exhibited a quantum yield of 12.3% (Crosby and Demas, 1971). Furthermore, the excited state of C-CNC is a double exponential decay process with an average fluorescence lifetime of 3.16 ns, demonstrating its excellent optical properties (Figure S6). In the control experiment, the nanocellulose did not show fluorescence enhanced with the ethanol concentration increased, which suggested the carboxyl groups were the luminance center of C-CNC (Figure S7).

In order to further understand the interactions of carboxyl groups on C-CNC, acid-base stability of C-CNC was also investigated at different pH solutions. Generally, C-CNC showed nice stability in an acidic environment (Figure S8a). However, it was found that the fluorescence intensity decreased slightly in an alkaline environment of C-CNC (Figure S8b). As shown in Figure S8c, the fluorescence intensity of C-CNC was highest at pH = 4, which may be ascribed to the through-space conjugation of carbonyl groups, especially hydrogen bonds increase, leading to the fluorescence enhancement. However, in basic environment, the interactions of carbonyl groups diminished due to the electrostatic repulsion, which resulted in reduced clustering of C-CNC. These results further verify the CTE mechanism of C-CNC.

**Figure 3:**
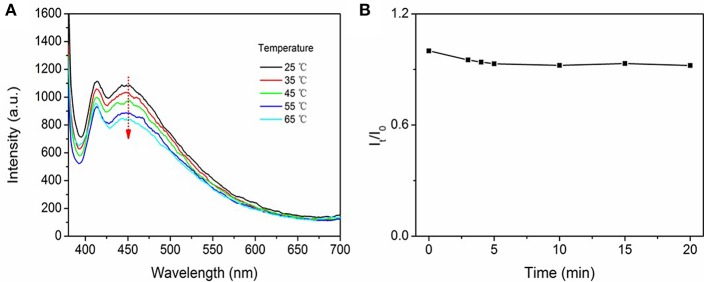
**(A)** Fluorescence of C-CNC (0.5 mg/mL) in water at different temperatures (25–65°C), (excitation wavelength = 360 nm), **(B)** Fluorescence intensity of C-CNC (excitation wavelength = 360 nm), I_t_ is fluorescence intensity after UV irradiation and I_0_ is fluorescence intensity without UV irradiation.

Subsequently, the emission of C-CNC was measured at different temperatures to determine the effect of temperature. When the temperature was raised from 25 to 65°C, a slight decrease was observed in fluorescence emission (Figure 3A). Additionally, the prepared C-CNC was measured by the DLS. It was found that the mean diameter of C-CNC at 25°C was 7.8 nm, however, the mean diameter was decreased to 6.2 nm at 65°C, indicating that the aggregates were reduced after heating (Figure S9). This slight change could be ascribed to the fact that an increase in temperature would disturb the interaction of the carbonyl groups, resulting in the interruption of C-CNC clusters, which is consistent with the literature (Ma et al., 2018). In addition, the photostability of C-CNC was examined. Compared with the traditional dyes, fluorescence materials with AIE properties generally show better photostability (Ma et al., 2018). After 20 min of UV irradiation, the fluorescence intensity of C-CNC decreased by about 8% (Figure 3B). These results demonstrate that C-CNC possess good anti-photobleaching ability, which is of great importance for further applications.

**Figure 4:**
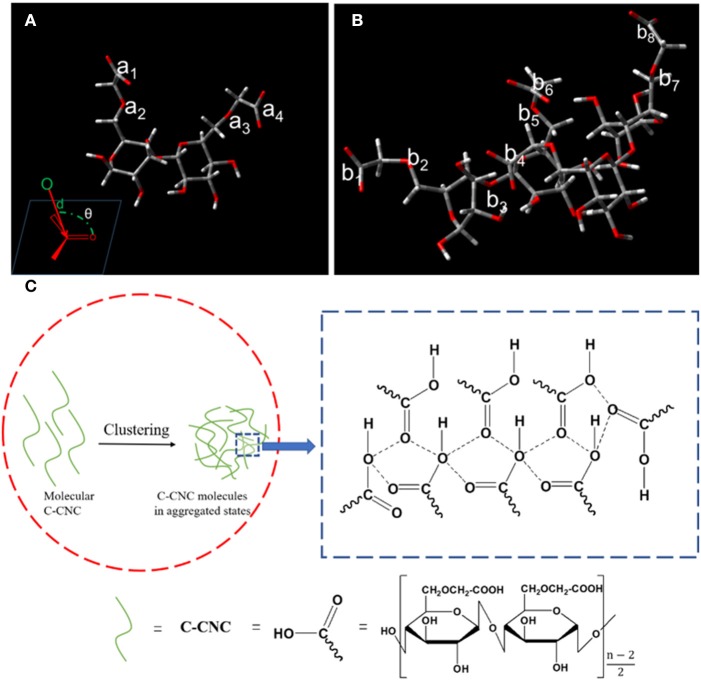
Optimized conformations of C-CNC **(A)**, inset: proposed model of n → π^*^ interaction and C-CNC dimer, **(B)** in water, red sticks = oxygen atoms, gray sticks = carbon atoms, **(C)** Schematic illustration of the emission mechanism of C-CNC.

To further decipher the emission mechanism, DFT calculations were used to investigate the clusteroluminescence. A two-molecule unit was used as the model for calculations. The configurations of C-CNC and C-CNC dimers in water were optimized using DFT, B3LYP/6-31G(d) (Figures 4A,B). The intramolecular distance (d) (a_1_-a_2_, a_3_-a_4_, Figure 4A, inset) of adjacent oxygen and carbonyl moieties was 2.54 Å and 2.54 Å, respectively. The Bürgi-Dunitz trajectory (a_1_-a_2_ and a_3_-a_4_) for nucleophilic addition (θ, Figure 4A, inset) was about 143° and 88° in individual C-CNC molecules. This indicated that the lone pair (n) on the oxygen atom did not effectively interpenetrate the empty π^*^ orbital of the carbonyl group and through-space conjugation did not occur (Feng et al., 2016; Wang et al., 2017). In Figure 4B, the DFT simulation illustrated that the intramolecular distance between adjacent oxygen and carbonyl moieties did not change a lot after formation of the C-CNC dimer. The intramolecular distance between b_1_-b_2_, b_3_-b_4_, b_5_-b_6_, b_7_-b_8_ was 2.54 Å, 2.47 Å, 2.50 Å and 2.50 Å, respectively. However, the Bürgi-Dunitz trajectory for nucleophilic addition (θ) changed a lot. The θ of b_1_-b_2_, b_3_-b_4_, b_5_-b_6_, b_7_-b_8_ was 100°, 113°, 119° and 111°, respectively. These results indicated a sub-van der Waals contact (d <3.22 Å), with the carbon of the acceptor carbonyl group along the Bürgi-Dunitz trajectory for nucleophilic addition (95° < θ <125°), which caused effective n → π^*^ interaction and through-space electronic interactions. As a result, LUMO orbitals were lowed. The theoretical calculations confirmed that clusteroluminescence can be attributed to through-space conjugation of oxygen atoms and carbonyl moieties caused by clustering of C-CNC (Figure 4C).

**Figure 5:**
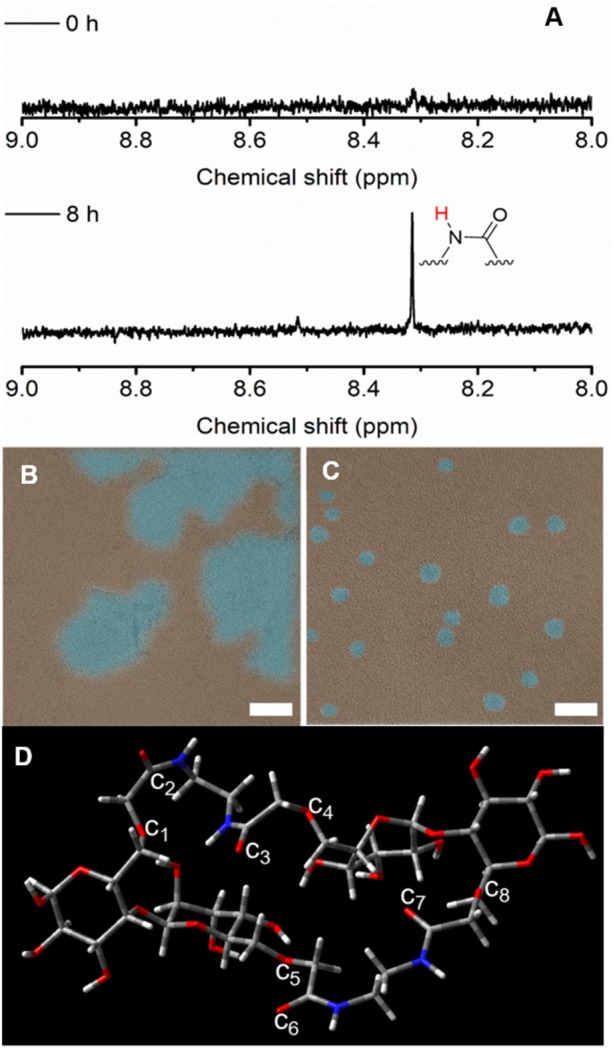
**(A)** Partial ^1^H NMR spectra of C-CNC and ethylenediamine in D_2_O before (top) and after (bottom) reaction, **(B)** TEM images of aqueous solution of C-CNC and ethylenediamine before and **(C)** after reaction, scale bars = 10 nm, **(D)** Optimized conformation of C-CNC/EDA, red sticks = oxygen atoms, gray sticks = carbon atoms.

Encouraged by this result, we continued our experiment to further confirm the interaction of carboxyl groups for the CTE phenomenon. In this experiment, we used ethylenediamine to react with carboxyl groups on C-CNC in water with different reaction time (Gómez et al., 2017). The prepared C-CNC/EDA was monitored by ^1^H NMR. In Figure 5A, it was found that no peaks were observed in the region of 8.2–8.4 ppm, but a new peak at ~8.32 ppm, which was attributed to amide units, indicating that C-CNC/EDA was successfully prepared. In a control experiment, ^1^H NMR of C-CNC did not show a change in the region of 8.2–8.4 ppm in the absence of ethylenediamine (Figure S10). TEM images before the reaction showed only dried random C-CNC aggregates and no nanodots (Figure 5B). After the reaction, TEM images of samples that had not undergone any separation or purification clearly showed nanodots with an average diameter of 5 nm (Figure 5C). This is further evidence for the amidation reaction between C-CNC and ethylenediamine. As expected, the fluorescence of C-CNC/EDA enhanced with the increase of reaction time compared with C-CNC and ethylenediamine (Figure S11). The chemical reactions occurred between the amino group of ethylenediamine and the carboxyl group of C-CNC, which were used to “lock” C-CNC, resulting in promoting the clustering of C-CNC. The results further demonstrate that the enhanced emission of C-CNC may be due to the interactions of carboxyl groups on C-CNC. To further verify the mechanism, DFT, B3LYP/6-31G(d) calculation was used. The distance (d) of c_1_-c_2_ c_3_-c_4_, c_5_-c_6_, and c_7_-c_8_ was 2.46 Å, 2.44 Å, 2.44 Å and 2.46 Å, respectively (Figure 5D). The Bürgi-Dunitz trajectory (θ) of c_1_-c_2_ c_3_-c_4_, c_5_-c_6_, and c_7_-c_8_ was 125°, 124°, 89° and 91°, respectively. Compared with the C-CNC cluster, it was surprisingly found that less O and carbonyl C couples formed effective n → π^*^ interaction in the C-CNC/EDA, which was inconsistent with our hypothesis. Therefore, such high fluorescence intensity may be attributed to the introduction of amide units, leading to through-space interaction of amide groups electrons resembling to those of aromatic structure caused by hydrogen bond (Feng et al., 2016). These results demonstrate that the enhanced fluorescence was caused by through-space conjugation of amide introduced. Furthermore, C-CNC was also investigated for its chiroptical properties. Interestingly, both C-CNC and C-CNC/EDA showed chirality (Figure S12), neither C-CNC nor C-CNC/EDA induced significant changes at detected concentrations, indicating that the chirality can be maintained very well after chemical modifications (Li et al., 2018a). The chirality of C-CNC could be further investigated for chiral sensing applications in future research.

## Conclusion

Biomass material, C-CNC, was used to prepare CTE nanomaterials via a chemical carboxylation method. The C-CNC had a good quantum yield of 7.8% and 12.3% in solid state and aqueous solutions, respectively. It also had a lifetime of 3.16 ns and showed excitation/polarity-dependent emission. Both experimental and DFT calculations have been employed to confirm that the clusteroluminescent emission originates from the intermolecular through-space conjugation between oxygen atoms and carbonyl groups on C-CNC. In addition, the prepared C-CNC/EDA also confirmed that the clusteroluminescence was attributed to the amide moieties and through-space conjugation between oxygen and carbonyl moieties. We believe our work not only provides an easy and green preparation of CTE nanomaterials, but gives a new possible mechanism of fluorescence emission of non-conjugated materials.

## Data Availability

The raw data supporting the conclusions of this manuscript will be made available by the authors, without undue reservation, to any qualified researcher.

## Author Contributions

ML designed the experiments, analyzed experimental results, and wrote the manuscript. XL and XA carried out the experiments. ZC and HX analyzed experimental results and provided guidance.

### Conflict of Interest Statement

The authors declare that the research was conducted in the absence of any commercial or financial relationships that could be construed as a potential conflict of interest.
