# Supplementary material for: Clustering-Triggered Emission of Carboxymethylated Nanocellulose
Source: Front Chem. 2019 Jun 20;7:447. doi: 10.3389/fchem.2019.00447 (PMC6596105; doi:10.3389/fchem.2019.00447)
Supplement: Supplementary file 1 [file Data_Sheet_1.docx]

Supplementary Material

Clustering-triggered Emission of Carboxymethylated Nanocellulose

Meng Li^1,2*^, Xiaoning Li^1,2^, Xuefei An^1,2^, Zhijun Chen^3*^, Huining Xiao^4*^

^1^Hebei Key Lab of Power Plant Flue Gas Multi-Pollutants Control, Department of Environmental Science and Engineering, North China Electric Power University, Baoding, 071003, PR China

^2^MOE Key Laboratory of Resources and Environmental Systems Optimization, Ministry of Education, Beijing, 102206, P. R. China

^3^Key Laboratory of Bio-based Material Science and Technology of Ministry of Education, Northeast Forestry University, Hexing Road 26, Harbin 150040, P. R. China

^4^Department of Chemical Engineering, University of New Brunswick, Fredericton, NB, Canada E3B 5A3

***Correspondence:**

Meng Li (M. L.), E-email address: [princessviola@163.com](mailto:princessviola@163.com)

# Supplementary experiment section

## Determination of carboxyl content of C-CNC

The carboxyl group content of C-CNC was determined using conductivity measurement. 0.3 g of dry C-CNC was added to 55 mL of deionized water, then 5 mL of 0.01 mol/L sodium chloride was added, and the pH was adjusted to 2.5-3.0 with 0.1 mol/L hydrochloric acid. 0.04 mol/L sodium hydroxide was added dropwise at a rate of 0.1 mL/min to gradually increase the pH to 11, meanwhile, measuring the conductance of the solution. The carboxyl content of C-CNC was determined by Eq (1):

$\mathbf{Q=}\frac{\boldsymbol{C\times(}\boldsymbol{V}_{\boldsymbol{2}}\boldsymbol{-}\boldsymbol{V}_{\boldsymbol{1}}\boldsymbol{)}}{\boldsymbol{m}}\boldsymbol{\times1000}$ (1)

Where Q is the carboxyl content (mmol/g), C is the sodium hydroxide concentration (mol/L), V_2_, V_1_ is the volume of sodium hydroxide solution in the conductivity change point, m is the weight of C-CNC.

## Fluorescent testing

The photoluminescence of the sample was measured by a fluorescence spectrophotometer with a voltage of 220 V and a slit for mid-range condition. In this paper, different excitation wavelengths, concentrations, different solvents, temperatures, *etc*. were tested.

## Photostability

2 mL of 5 mg/mL C-CNC aqueous solution was added into a cuvette and placed a thermostat that had been pre-set at a temperature (25-65 °C) for 60 min, then taken out for measuring with fluorescence spectrophotometer immediately (excitation wavelength = 360 nm).

## Quantum Yield Determination

The relative quantum yields of C-CNC were measured in reference to quinine sulfate. The same excitation wavelength, gain and slit bandwidth were applied to both samples. The formula for QY measurement was determined as follows (Crosby and Demas, 1971):

**φ = φ_st_(K/K_st_)(η/η_st_)^2^**

Where φ is the quantum yields, K is the slope determined by the curves, η is the refractive index. The φ_st_, K_st_, η_st_ are the parameters of quinine sulfate. For these aqueous solutions, η/η_st_ = 1.

## Time resolved photoluminescence

The time-correlated photoluminescence (PL) signals of C-CNC in solution and on electrode were recorded by FLS980 Spectrometer. The PL decay signals were detected at 450 nm upon excitation with picosecond pulsed diode laser EPL-450 at 360 nm.

## Dynamic light scattering (DLS) Determination

C-CNC (50 mg) was dissolved in 90% ethanol solutions (100 mL) and mixed uniformly. 2 mL solutions were added into a cuvette and placed a thermostat that had been pre-set at a temperature (25 °C and 65 °C) for 3 min, then the mean sizes of C-CNC were determined by DLS.

# Supplementary Figures





**Figure S1.** [Carboxyl](javascript:;) [group](javascript:;) [content](javascript:;) of C-CNC.


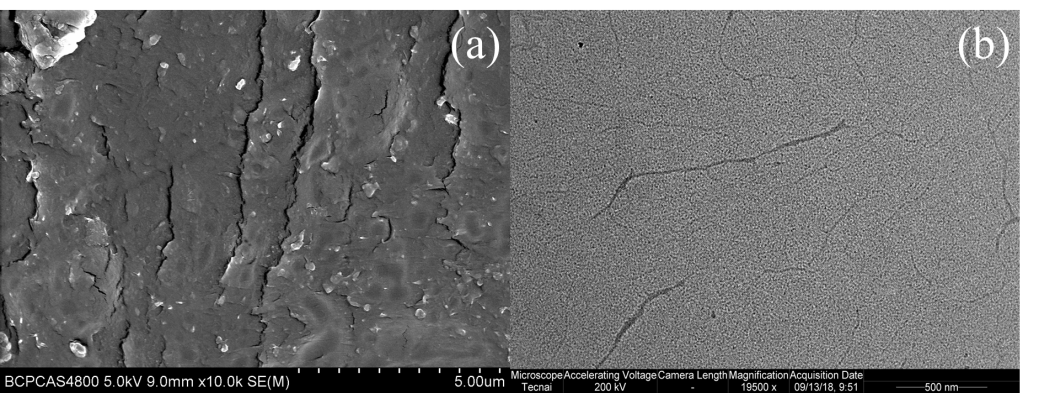


**Figure S2.** **(a)** SEM images of C-CNC, scale bars = 5.00 um, **(b)** TEM images of C-CNC, scale bars = 500 nm.





**Figure S3.** Raman spectra of C-CNC.





**Figure S4.** Fluorescence emission of C-CNC (0.5 mg/mL) in water at different excitation wavelengths.


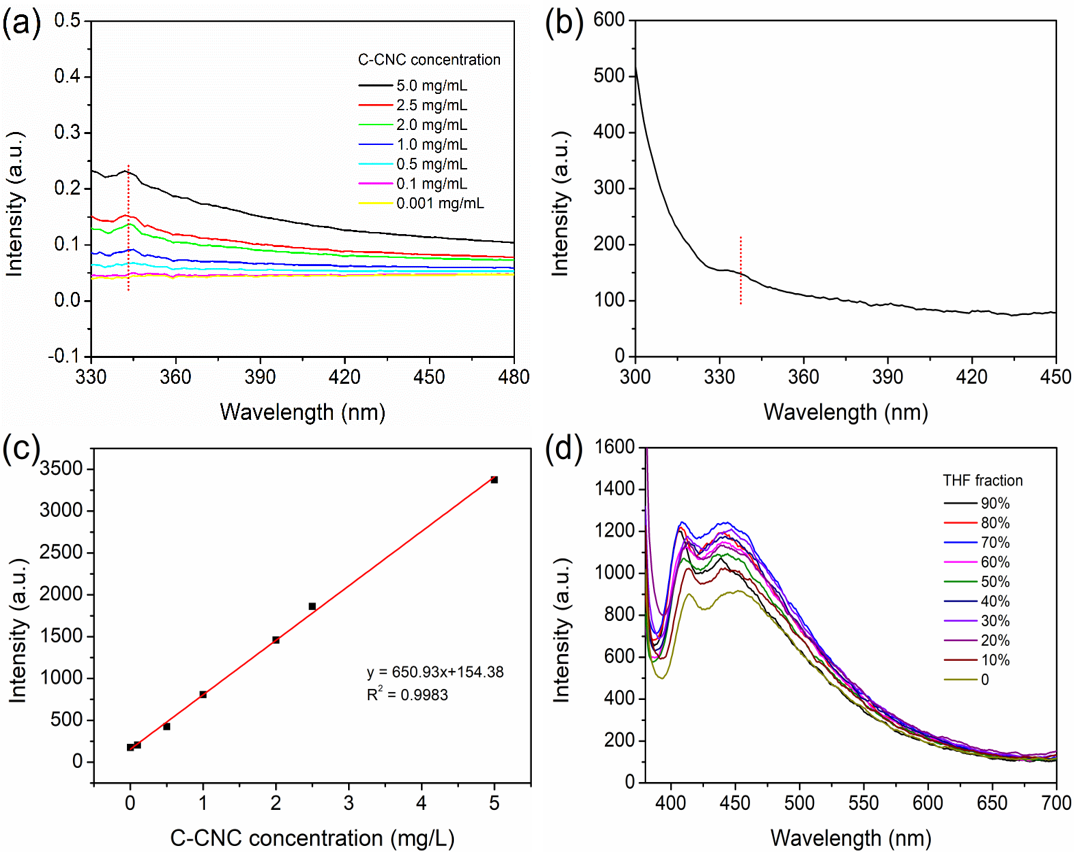


**Figure S5. (a)** UV-vis spectra of C-CNC in 50% ethanol solutions at different concentrations (0.001-5.00 mg/mL), **(b)** Excitation spectra of C-CNC solutions (0.5 mg/mL), **(c)** Fluorescence emission of C-CNC at different concentrations in water (excitation wavelength = 360 nm), **(d)** Fluorescence emission of C-CNC (0.5 mg/mL) in mixtures of THF and water (excitation wavelength = 360 nm).





**Figure S6.** Emission decay profiles of C-CNC solutions (excitation wavelength = 360 nm).





**Figure S7.** Fluorescence emission of nanocellulose (0.5 mg/mL) in mixtures of ethanol and water (excitation wavelength = 360 nm).


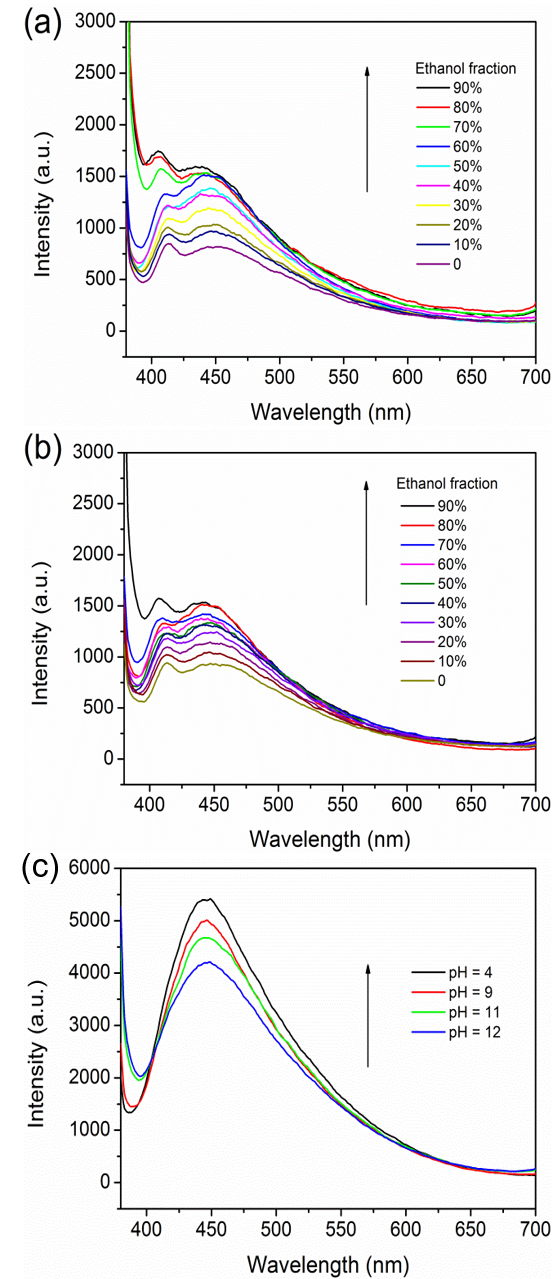


**Figure S8.** **(a)** Fluorescence emission of C-CNC (0.5 mg/mL) in mixtures of ethanol (pH = 4) and water (excitation wavelength = 360 nm), **(b)** Fluorescence emission of C-CNC (0.5 mg/mL) in mixtures of ethanol (pH = 9) and water (excitation wavelength = 360 nm), **(c)** Fluorescence emission of C-CNC (5.0 mg/mL) in water solutions at different pH (excitation wavelength = 360 nm).


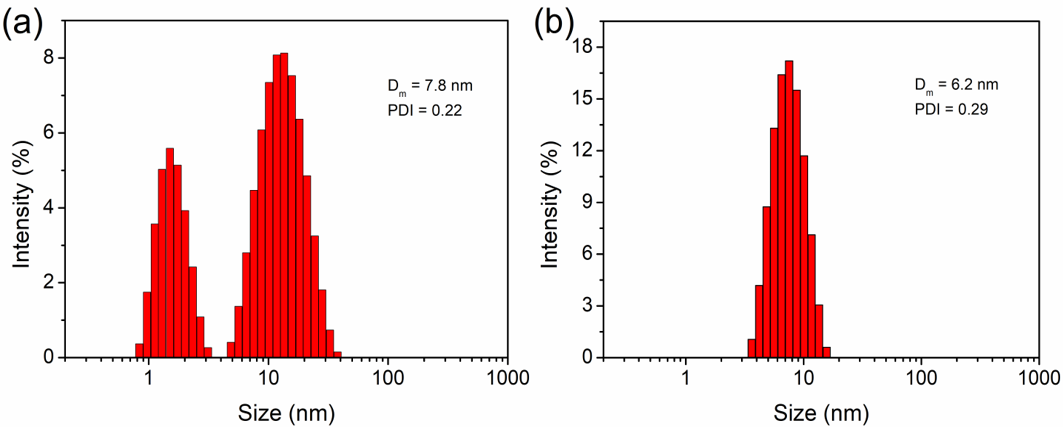


**Figure S9. (a)** DLS data of C-CNC in 90% ethanol solutions at 25℃, **(b)** DLS data of C-CNC in 90% ethanol solutions at 65℃ (D_m_ = mean diameter, PDI = polydispersity index).

**Figure S10.** ^1^H NMR spectra of C-CNC in D_2_O before and after reaction at 100 °C.





**Figure S11.** Fluorescence emission of C-CNC (0.5 mg/mL) with low temperature hydrothermal reaction (excitation wavelength = 360 nm).





**Figure S12.** CD spectra of C-CNC and C-CNC/EDA with different reaction time (0.02 mg/mL).

**References**

Crosby, G. A., and Demas, J. N. (1971). Measurement of photoluminescence quantum yields. Review. *J. Phys. Chem.* 75**,** 991–1024. doi: 10.1021/j100678a001.
